# Supplementary material for: Enhancing generalizability in classification of peripheral neural recordings with graph neural network
Source: PLoS One. 2026 Apr 17;21(4):e0345204. doi: 10.1371/journal.pone.0345204 (PMC13089695; doi:10.1371/journal.pone.0345204)
Supplement: Attachment — Submitted filename: R2_Response_to_Reviewers.pdf [file pone.0345204.s002.pdf]

We sincerely thank the editor and reviewers for their time in reviewing the revised manuscript, and greatly appreciate the constructive feedback provided. We have considered each comment. Responses to each reviewer comment are provided in blue text throughout this letter.

Reviewer #1: I would like to thank the authors for their comprehensive responses and the revisions made to the manuscript. The clarifications provided are exhaustive and satisfactorily address my previous concerns regarding the state-of-the-art comparison and the methodological details.
I have just one minor note regarding the presentation of the results, which is a suggestion and not a mandatory requirement for acceptance. While I appreciate the inclusion of the network topology figures, I believe that the quantitative results currently presented in Tables 2, 3, and 4 would be much more effective if visualized as charts (e.g., grouped bar plots with error bars). Visualizing the data in this way would allow readers to immediately grasp the performance trends and the stability of the model across subjects, whereas large tables can be harder to interpret at a glance. The detailed numerical tables could then be moved to the Supplementary Material.
However, I leave this decision entirely to the authors' discretion. I am satisfied with the scientific content and the improvements made, and I support the publication of the manuscript in its current form.

We sincerely thank the reviewer for their kind remarks and thoughtful feedback on the revised manuscript. We appreciate the suggestion to visualize the quantitative results in Tables 2, 3, and 4 using grouped bar plots with error bars. After careful consideration, we chose to retain the tables in the main text because a few of the reported differences between models may be less clearly distinguishable when visualized in plot form, and we believe that presenting the exact numerical values allows for more precise comparison. We are grateful for the reviewer’s support and constructive suggestions that have helped improve the quality and clarity of the manuscript.

Reviewer #3: All my comments have been addressed properly. I don't have further questions. The comparison between geodesic-based and the Euclidean graph looks reasonable, which I think is the key of this paper.

We sincerely thank the reviewer for their time and thoughtful feedback on the revised manuscript. We greatly appreciate the careful evaluation and are pleased that the comparison between geodesic-based and Euclidean graph constructions is now clear and reasonable. The reviewer’s comments have been instrumental in strengthening the manuscript, and we are grateful for their support.
